# The time course and mechanisms of change in biomarkers of joint metabolism in response to acute exercise and chronic training in physiologic and pathological conditions

**DOI:** 10.1007/s00421-019-04232-4

**Published:** 2019-10-24

**Authors:** Harry M. Roberts, Rebecca-Jane Law, Jeanette M. Thom

**Affiliations:** 1 School of Biosciences and Medicine, University of Surrey, The Leggett Building, Daphne Jackson Road, Guildford, GU2 7WG UK; 2 North Wales Centre for Primary Care Research, School of Health Sciences, Bangor University, Bangor, UK; 3 School of Medical Sciences, University of New South Wales, Sydney, Australia; 4 School of Sport, Health and Exercise Sciences, Bangor University, Bangor, UK

**Keywords:** COMP, Arthritis, Walking, Running, Knee injury, Bone

## Abstract

**Purpose:**

The benefits of exercise across the lifespan and for a wide spectrum of health and diseases are well known. However, there remains less clarity as to the effects of both acute and chronic exercise on joint health. Serum biomarkers of joint metabolism are sensitive to change and have the potential to differentiate between normal and adverse adaptations to acute and chronic load. Therefore, the primary objective of this review is to evaluate how serum biomarkers can inform our understanding of how exercise affects joint metabolism.

**Methods:**

A comprehensive literature search was completed to identify joint biomarkers previously used to investigate acute and chronic exercise training.

**Results:**

Identified biomarkers included those related to joint cartilage, bone, synovium, synovial fluid, and inflammation. However, current research has largely focused on the response of serum cartilage oligomeric matrix protein (COMP) to acute loading in healthy young individuals. Studies demonstrate how acute loading transiently increases serum COMP (i.e., cartilage metabolism), which is mostly dependent on the duration of exercise. This response does not appear to be associated with any lasting deleterious changes, cartilage degradation, or osteoarthritis.

**Conclusion:**

Several promising biomarkers for assessing joint metabolism exist and may in future enhance our understanding of the physiological response to acute and chronic exercise. Defining ‘normal’ and ‘abnormal’ biomarker responses to exercise and methodological standardisation would greatly improve the potential of research in this area to understand mechanisms and inform practice.

## Introduction

In recent years, there has been a greater understanding of the benefits of exercise across the lifespan and continuum of health and disease. The focus of much of this research has been the benefits of exercise on the cardiovascular and muscular systems. However, there is currently less clarity of the effects of exercise on the structures comprising the joints. Our knowledge is confounded by potential contrasting effects of regular moderate joint-loading exercise versus exercise that ‘overloads’ joints, as well as exercise in people with joint injury or exercise for people with joint diseases such as osteoarthritis or rheumatoid arthritis. For example, 20–50% of patients with anterior cruciate ligament damage will have evidence of osteoarthritis within the next 10–20 years (Sepulveda et al. 2017). In addition, people that undertake heavy physical workloads and/or frequent exposure to biomechanical stressors of the joint (e.g., squatting and kneeling) in their occupation over many years have been found to be at higher risk of osteoarthritis than those in other occupations (Yucesoy et al. 2015). Confounding this is the fact that exercise has been utilised as a beneficial treatment for people with osteoarthritis and rheumatoid arthritis as well as in recovery from joint injury (Cooney et al. 2011; Beckwée et al. 2013; McAlindon et al. 2014; Metsios et al. 2015; Duncan et al. 2016; Filardo et al. 2016; Vannini et al. 2016; Bartholdy et al. 2017; Sepulveda et al. 2017). Therefore, to date, there is still confusion in the general public, in addition to within health professionals, as to the benefits of exercise on the health of joints.

There is a growing burden of musculoskeletal disease in our society, leading to extensive health costs (Yucesoy et al. 2015) [e.g., knee osteoarthritis prevalence of approx. 10% in adults 60+ years and total treatment costs of 1–2.5% of the gross domestic product for westernised countries (Rovati et al. 2013)]. Therefore, the previous exercise-related research on joints has focused on diseases such as osteoarthritis and to a lesser extent rheumatoid arthritis. Part of this body of research has been to determine if there are specific biochemical markers (biomarkers: predominantly from serum) that can be used as a clinical tool for prevention, assessment, and treatment of joint disease. Currently, there are no clear biomarkers for osteoarthritis, though there has been interest in determining the biomarker response to joint loading as a possible measure of joint health and in the prediction of early osteoarthritis (Cattano et al. 2017b). Due to the conflicting information mentioned above regarding how exercise interrelates with joint health, the need for joint biomarkers that can differentiate between normal load adaptations and those of an adverse load adaptation is highly desirable. Thus, there is a need to understand how exercise interrelates with disease and joint health in general. Therefore, the aim of this review is to provide an overview of joint physiology and evaluate the most promising serum joint biomarkers, including those related to cartilage, bone, the synovium, and joint inflammation. This review will detail and discuss how these serum biomarkers respond to acute joint loading and chronic exercise training, both in healthy individuals, and among individuals with pathological joint conditions such as osteoarthritis and rheumatoid arthritis. Of particular interest is the effect of exercise duration, the modality of exercise, as well as the possible mechanisms and pathways of change. Understanding the biomarker response to exercise may ultimately lead to better treatment and prevention of joint diseases.

## Overview of joint physiology

**Fig. 1:**
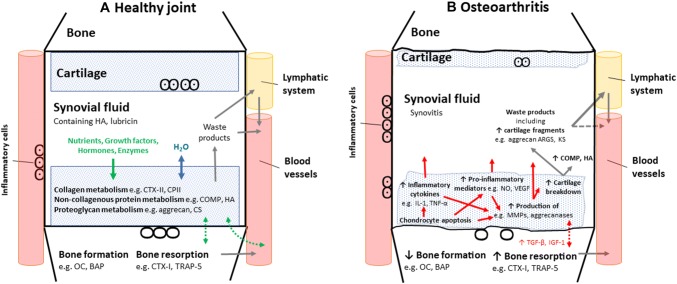
Schematic of **a** healthy synovial joint and **b** synovial joint highlighting changes following osteoarthritis with alterations in key potential serum biomarkers from cartilage, synovial fluid, and/or bone. *BAP* bone alkaline phosphatase, *COMP* cartilage oligomeric matrix protein, *CPII* C propeptide of type II collagen, *CS* chondroitin sulfate, *CTX-1* C-telopeptide of type I collagen, *CTX-11* C-telopeptide of type II collagen, *H*_*2*_*O* water, *HA* hyaluronan, *IGF-1* insulin-like growth factor, *IL-1* interleukin-1, *KS* keratan sulfate, *MMPs* matrix metalloproteinases, *NO* nitric oxide, *OC* osteocalcin, *TGF-β* transforming growth factor beta, *TNF-α* tumor necrosis factor, *TRAP-5* tartrate-resistant acid phosphatase 5b, *VEGF* vascular endothelial growth factor

For joints to function effectively under normal loading conditions and for overall joint health, the main structures, including the joint cartilage, subchondral bone and the synovium, need to work in combination, as schematically represented in Fig. 1 (healthy versus what may occur with joint degradation). In addition to this, muscles, ligaments, tendons, and systemic effectors may also be important in overall joint health. It is our understanding that the molecular balance between anabolic and catabolic activity may be key in the maintenance of joint integrity as well as the ability to provide nutrients to and remove waste products from the joint due to repeated mechanical loading, via blood and lymph vessels.

Articular cartilage predominantly consists of chondrocytes, with the main cartilage volume being occupied by an extracellular matrix containing a dense network of collagen fibrils (predominantly type II) and proteoglycan molecules (Yamaguchi et al. 2013). The main proteoglycan is aggrecan. Aggrecan contains keratan sulfate and chondroitin sulfate, which are attached to hyaluronan and is stabilised by link protein. Whereas collagen provides the tensile strength to cartilage, the proteoglycans attract water into the cartilage and this swelling pressure resists compressive loads and minimises deformation. Overall cartilage metabolism can be attributed to the balance between the anabolic and catabolic activities of chondrocytes (Yamaguchi et al. 2013). Chondrocytes produce various cytokines, chemokines, proteases, and inflammatory mediators that promote the deterioration of articular cartilage (Fig. 1b). These include the production of matrix metalloproteinases (MMPs), collagenases, a disintegrin metalloproteinase with thrombospondin motifs (ADAMTS), and aggrecanases as well as cytokines, such as interleukins (e.g., IL-1, IL-6, and IL-8), tumor necrosis factor (TNF-α), and nitric oxide (Abramson and Krasnokutsky 2006; Yamaguchi et al. 2013). Collagen metabolism can be measured from the ratio of circulating levels’ markers of collagen breakdown to synthesis measured from cleavage fragments [e.g., the ratio of collagen type II cleavage product (C2C) to C propeptide of type II collagen (CPII)] (Yamaguchi et al. 2013).

The synovium, that contains blood, nerve and lymphatic vessels, is a deformable, non-adherent tissue surface that provides lubrication of cartilage and controls the synovial fluid volume and the nutrition of chondrocytes within joints (Smith 2011). Hyaluronan is a key component of synovial fluid, providing synovial fluid viscosity and volume, and thus ‘cushioning’, as well as lubrication (Smith 2011). Hyaluronan is thought to be the main factor behind retaining constant synovial fluid volume during exercise (Levick and McDonald 1995). Hyaluronan is also found in different molecular mass forms, with higher mass forms providing more lubrication. With ageing, a shift to hyaluronan of lower mass forms as well as a decrease in the amount of hyaluronan (independent of presence of osteoarthritis) has been observed, thus being a potential link to a decrease synovial fluid lubricant quality with age (Temple-wong et al. 2016). Lubrication of the cartilage surface is also facilitated by glycoproteins, especially lubricin [also known as superficial zone protein, proteoglycan-4 (PRG-4)] due to its localisation to the surface of both synovium and cartilage (Jay and Waller 2014).

The normal function of synovial joints is to provide cushioning and lubrication during movement, and this movement assists in the healthy function and nutrition of the joint. Cartilage is avascular; however, diffusion between cartilage and synovium (and between cartilage and bone) is increased by cyclic loading. Molecules, such as essential nutrients, growth factors, enzymes, and waste products, can enter and/or exit cartilage via the synovium and subchondral bone (Findlay and Kuliwaba 2016). Though, it is thought that the synovium provides the major route to deliver nutrition for chondrocytes. Subchondral bone may contribute to nutrition in immature joints, but in adult joints, this route is unlikely to be significant (Smith 2011). Flow directly between synovial fluid and plasma may be limited by the size of molecules, whereas larger molecules, such as proteins, may leave the synovial fluid through lymphatic vessels, a process that is not size selective. The time course for these molecules to enter the blood stream following exercise is not well understood, which then has implications on the effectiveness of these as biomarkers when measured directly after acute exercise.

The bones adjacent to joints are key in the transmission of load during locomotion and physical activities. Moreover, joint loading is also a key regulator of bone health, including the maintenance of bone mineral density, along with systemic hormones and local factors (e.g., cytokines and growth factors) (Hlaing and Compston 2014). This is highlighted by the importance of joint loading for conditions such as menopause and osteoporosis (Harding and Beck 2017). Similarly, treatment, e.g., hormone treatment for cancer, can adversely affect bone mineral density (Dalla Via et al. 2018). Bone metabolism involves several metabolic processes, i.e., the combination or counteraction of the metabolic process of bone formation (anabolic), and the metabolic process of bone resorption (catabolic) (Hlaing and Compston 2014). Ultimately, it is these two processes that are key in the regulation of bone mass, as well the principle functions of the skeleton, including the mechanical support of the body, calcium deposition, and haemopoiesis. Unfortunately, a disruption in the bone anabolic–catabolic milieu, often a consequence of changes in the controlling factors, can lead to the onset of metabolic joint disease such as osteoporosis.

It is thus believed that moderate mechanical loading maintains the integrity of articular cartilage and the joint per se by aiding the movement of nutrients and waste products between structures in the joint (Sun 2010). In addition, moderate physical activity in humans seems to be protective against osteoarthritis and is used as a therapy for people with existing osteoarthritis (Beckwée et al. 2013; McAlindon et al. 2014). However, both disuse and overuse are thought to result in cartilage degradation (Sun 2010; Nomura et al. 2017; Campbell et al. 2018). Osteoarthritis has multiple and variable causes; however, exercise alone has rarely been associated with increased risk of osteoarthritis. Instead, many specific mechanical (e.g., gait mechanics) and biological (e.g., loss of cartilage and inflammation) factors may combine with other risk factors (sedentarianism, obesity, genetics, injury, and ageing) and precede the onset of osteoarthritis (Edd et al. 2017). Therefore, understanding the joint’s response to exercise, and how we can measure this, is vital for overall joint health and further understanding of joint diseases.

## The most promising serum joint biomarkers

**Fig. 2:**
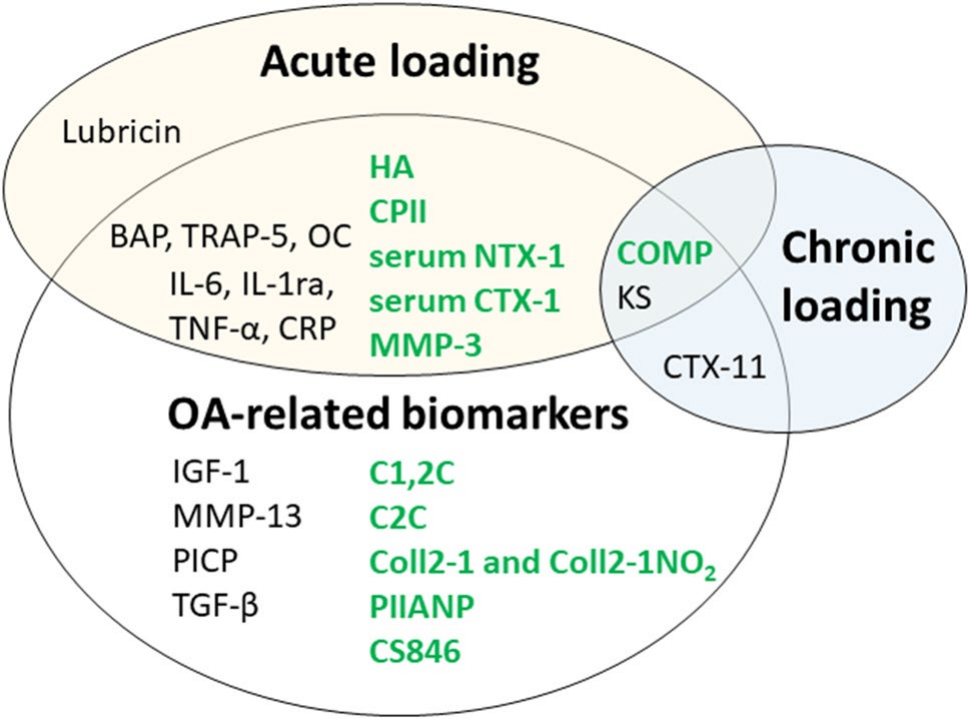
Promising biomarkers previously used to investigate acute and chronic joint loading. All biomarkers are serum unless stated. Green OARSI recommended osteoarthritis-related biomarkers (Kraus et al. 2017a, b). *BAP* bone alkaline phosphatase, *C1,2C* Col2-3/ 4 C-terminal cleavage product of types I and II collagen, *C2C* Collagen type II cleavage product, *Coll2-1 *and* Coll2-1 NO*_*2*_ nitrated epitope of the α-helical region of type II collagen, *COMP* cartilage oligomeric matrix protein, *CPII* C propeptide of type II collagen, *CRP* C-reactive protein, *CS846* chondroitin sulfate 846 epitope, *CTX-1* C-telopeptide of type I collagen, *CTX-11* C-telopeptide of type II collagen, *HA* hyaluronan, *IGF-1* insulin-like growth factor, *IL* interleukin, *KS* keratan sulfate, *MMP* matrix metalloproteinase, *NTX-1* N-telopeptide of type I collagen, *OC* osteocalcin, *PICP* procollagen type I C propeptide, *PIIANP* N propeptide of collagen IIA, *TGF-β* transforming growth factor beta, *TNF-α* tumor necrosis factor, *TRAP-5* tartrate-resistant acid phosphatase 5b

Biomarkers in the blood offer a promising alternative to traditional methods to assess the health of joints. Serum biomarkers are accessible and may offer sensitive methods to monitor early joint adaptation, degenerative change, and osteoarthritis (Bauer et al. 2006). Biomarker research is particularly attractive, given that clinical measures of early knee joint adaptation are elusive. Traditional methods of joint assessment include using X-ray with Larsen scores forming a key outcome variable for research studies. However, there are limitations associated with X-rays, as they change slowly in most people, with 6 months to a year often needed to capture changes in an individual patient (Sokka 2008). There are also safety implications associated with radiation. Additional methods to assess the joint include magnetic resonance imaging (MRI) and sonography. MRI enables high quality soft tissue contrast, quantitative measurement of tissues and has been used to explore acute and chronic adaptation to training; however, it is expensive, time consuming and requires significant expertise. High-resolution sonography is a relatively simple technique, quick, and inexpensive compared to MRI, and it also provides real-time imaging and immediate feedback. Sonography has also been successfully used to explore the effects of acute exercise (Harkey et al. 2017); however, it does not reliably provide the level of measurement precision required to explore changes in cartilage thickness following acute exercise (Roberts et al. 2016). In contrast to X-ray, MRI, and sonography, serum biomarkers have been shown to provide a more sensitive method of monitoring changes. Moreover, while this research is still progressing, findings have demonstrated a relationship between serum biomarkers and traditional measures; for example, the response of serum cartilage oligomeric matrix protein (COMP) to exercise is understood to relate to an increase in cartilage metabolism (Neidhart et al. 2000) and has been correlated independently with decreases in cartilage volume (Kersting et al. 2005) and changes in cartilage thickness over a 5-year period (Erhart-Hledik et al. 2012). These biomarkers include molecules or molecular fragments from the extracellular matrix or cellular metabolism of the articular cartilage, subchondral bone and synovial tissue. Several inflammatory biomarkers, including cytokines, chemokines, as well as signalling molecules and growth factors may also be potential markers. To date, biochemical markers have been typically utilised to diagnose osteoarthritis, to assess and classify the burden of disease, as a prognostic tool, and to investigate efficacy of interventions (Bauer et al. 2006). Studies have also explored the effect of acute and chronic loading on biomarkers. These investigations have been completed for several reasons: to help improve the use of biomarkers as clinical tools and improve biomarker sensitivity; to monitor and assess the load and thus the impact of physical activity on the joint and to explore the effects of exercise on healthy, injured, and degenerative joints. This is of particular importance, given that exercise is prescribed as both as a preventative and rehabilitative tool for the knee joint. Unfortunately, a number of commonly identified osteoarthritis biomarkers have not been fully explored with regards to acute and chronic mechanical loading, as highlighted in Fig. 2. The biomarkers that have commonly been investigated in response to acute and chronic loading are documented in Table 1. These include biomarkers of several joint tissues, including cartilage, synovium and bone. These biomarkers are typically associated with joint tissue synthesis, degradation, or metabolism and can be assessed in body fluid such as synovial fluid, serum and urine. Serum biomarkers are thought to be the most practical, as are easily accessible to people with both healthy and inflamed joints and are thus the focus of this review. However, it is also important not to confuse some potential serum biomarkers that may denote joint disease with markers that are associated with the normal systemic response to fatiguing exercise, e.g., markers of inflammation or creatine phosphokinase. It is also not clear what may be the systemic effects of exercise on joint homeostasis per se, i.e., if there is any contribution of biomarkers released from muscles, ligaments or tendons in addition to what is released from joints in levels observed in serum following exercise (Sun 2010).

**Table 1:** Summary of common serum biomarkers measured following acute exercise loading or at rest after chronic exercise loading

	Acute loading*			Chronic loading†
	Short, e.g., 30 min walking	Medium length > 30–60 min	Prolonged exercise > 60 min—ultramarathon	Exercise training/athletes
Biomarkers from cartilage and/or synovial fluid	COMP ↑ HA ↔ ↑ Lubricin ↑ MMPs↑	COMP ↑	COMP ↑ HA ↔ ↓ MMPs↑	COMP ↔ ↑ MMPs ↔ ↑ C2C, CPII, C2C:CPII ↔ CTX-II ↓
	IL-10 (Anti-inflammatory) ↑		IL-1ra ↑, ↑** IL-6, TNF-α ↑ CRP ↑, ↑**	IL-1b ↔ IL-6 ↔ ↓ IL-8 ↔ ↓ TNF-α ↔ ↓ CRP ↔ ↓
Biomarkers from bone	Formation: PICP ↔ ↓** BAP ↔ ↑ OC ↔	Formation: BAP ↔ ↑ OC ↔ Resorption: CTX-1 ↑** NTX-1 ↑** TRAP-5 ↔ ↓**	Formation: BAP ↔ ↓** OC ↔ PICP↓, ↑** Resorption: TRAP-5 ↑ ITCP ↑ ↔	Formation: BAP, OC, PICP ↑ Resorption: Pyridinoline ↔ ↑ Deoxypyridinoline ↑ CTX-1 ↔ NTX-1 ↓ TRAP-5 ↑

Biomarker: * measured immediately post-acute exercise unless specified, ** measured hours/days following exercise, † measured at rest following chronic exercise training. ↑ increase, ↓ decrease, ↔ no change

*BAP* bone alkaline phosphatase, *C2C* Collagen type II cleavage product, *COMP* cartilage oligomeric matrix protein, *CPII* C propeptide of type II collagen, *CRP* C-reactive protein, *CTX-1* C-telopeptide of type I collagen, *CTX-11* C-telopeptide of type II collagen, *HA* hyaluronan, *IL* interleukin, *ICTP* cross-linked carboxyterminal telopeptide of type I collagen, *MMPs* matrix metalloproteinases, *NTX-1 *N-telopeptide of type I collagen, *OC* osteocalcin, *PICP* procollagen type I C propeptide, *TNF-α* tumor necrosis factor, *TRAP-5* tartrate-resistant acid phosphatase 5b

## Structural biomarkers, including biomarkers related to cartilage, bone, and synovial fluid

Structural molecules of the joint, or fragments thereof, are understood to be the most promising biomarkers for use following acute and chronic exercise as well as with joint degradation. These biomarkers are specific to joints and generally have higher serum levels following increased cartilage breakdown and/or metabolism of the cartilage. Promising cartilage biomarkers include those of collagen metabolism [(e.g., C-telopeptide of type II collagen (CTX-11), CPII], non-collagenous protein metabolism [(e.g., COMP, hyaluronan)], and proteoglycan metabolism (e.g., MMP-3, aggrecan ARGS), for overview refer to Figs. 1 and 2. Biomarkers of Type II collagen may be ideal markers of cartilage health as Type II collagen is the most abundant protein in cartilage, it is relatively specific to articular cartilage and the turnover is normally slow (Birmingham et al. 2007). In terms of biomarkers of synovial fluid, hyaluronan and lubricin have both been previously used.

Interest in potential biomarkers from subchondral bone, to which cartilage is attached, has centred on bone metabolism, either enzymes or degradation products (bone matrix proteoglycans and glycoproteins), as well as inflammatory markers that are released into the circulation, reflecting the bone remodelling processes. Several blood biomarkers have previously been investigated to explore bone metabolism (Hlaing and Compston 2014). Key serum markers of bone formation include osteocalcin and Bone Alkaline Phosphatase (BAP). In contrast, C-telopeptide of type I collagen (CTX-1), N-telopeptide of type I collagen (NTX-1), and Tartrate-resistant acid phosphatase 5b (TRAP-5) are key markers of bone reabsorption. These bone turnover biomarkers can be used to estimate the direction of bone metabolism and potentially to assess how both acute and chronic loading can influence bone health. Bone metabolism markers have potential clinical utility and are reported to be associated with bone microarchitecture in older adults and to predict fracture risk (Sarkar et al. 2004; Hlaing and Compston 2014). The use of bone metabolism markers as markers specific to joint health following acute and chronic exercise has so far been limited. However, these markers may have utility and this will be discussed.

## Biomarkers used to investigate pathological conditions of the joint

One of the quandaries in determining appropriate biomarkers of exercise on joint metabolism is whether markers of osteoarthritis can be used. This is because biomarkers of osteoarthritis may only be elevated or lowered due to the disease condition and may not alter under acute changes due to loading and, therefore, do not show exercise-induced change per se. This is especially important in attempting to determine the chronic effect that overloading on joints may have in joints at risk of developing osteoarthritis over and above the disease progression itself. In addition to this, if ‘underloading’, i.e., being sedentary is also linked to developing osteoarthritis, perhaps this also influences the expression of osteoarthritis joint biomarkers? Studies investigating joint underloading have observed thinning of articular cartilage, with decreases in COMP, proteoglycan content, and hyaluronan, though collagen was found more resistant to change (Vanwanseele et al. 2002; Liphardt et al. 2009, 2015; Nomura et al. 2017). Most unloading adaptations return to normal when loading resumes, especially from non-rigid unloading. However, long-term rigid immobilisation in rats have observed replacement of cartilage by bone via chondral vascularisation, thus suggesting irreversible degradation can occur (Campbell et al. 2018). The other important consideration in the effect of joint injury on future osteoarthritis progression and what affects this may have to the serum biomarker concentrations (Luyten et al. 2018).

Osteoarthritis is a heterogeneous, degenerative, low-grade inflammatory joint disease which affects all articular tissues as well as periarticular tissues (e.g., tendons, adipose tissue, and muscles) and is predominantly found in older adults (Lee et al. 2013; Henrotin et al. 2016; Mobasheri et al. 2017). Osteoarthritis joints are characterised by joint damage and resultant altered biomechanics (Luyten et al. 2018). Traditional classification of osteoarthritis predominantly relies on clinical features such as disability and pain as well as joint degeneration from radiographs, with multiple prognostic factors and a variety of classification criteria (Creamer 2000; Hiligsmann et al. 2013; Luyten et al. 2018). Currently, the classification of early or established osteoarthritis does not include serum biomarkers (Altman et al. 1986; Mobasheri et al. 2017; Luyten et al. 2018). However, many potential biomarkers have been considered for osteoarthritis, as there is a need for biomarkers to assist in disease diagnosis, and as a more sensitive method to detect disease progression and assessment (Bauer et al. 2006; Mobasheri et al. 2017; Bay-Jensen et al. 2018). Possible osteoarthritis biomarkers could be broadly grouped into (1) products of bone and cartilage degradation and (2) pro- and anti-inflammatory agents (Abramson and Krasnokutsky 2006; Mabey and Honsawek 2015). Cartilage degradation biomarkers include CTX-11, COMP, a number of MMPs (e.g., MMP-3, MMP-13), and collagen and aggrecan specific biomarkers, e.g., N propeptide of collagen IIA (PIIANP) (Abramson and Krasnokutsky 2006). For example, an increased concentration of aggrecan fragments in the synovial fluid has been observed in osteoarthritis patients, which are thought to reflect increased cartilage degradation (Roughley and Mort 2014). Serum hyaluronan is also increased with osteoarthritis as well as several subchondral bone biomarkers that are linked to increased osteophyte formation, e.g., CTX-1, NTX-1, and TRAP-5. The bone biomarkers that have been linked to osteoarthritis may result from local production of anabolic factors including transforming growth factor beta (TGF-β) and insulin-like growth factor (IGF-1), see Fig. 1 (Abramson and Krasnokutsky 2006). An array of increased inflammatory markers has also been reported in osteoarthritis which include several interleukins, vascular endothelial growth factor (VEGF), C-reactive protein (CRP), TNF-α, nitric oxide, prostaglandins, and leukotrienes and their presence may accelerate the deterioration of cartilage (Abramson and Krasnokutsky 2006). The observation that chondrocyte apoptosis may precede cartilage matrix damage (Sun 2010) suggests that alterations in cellular metabolism contribute to the onset and progression of osteoarthritis (Yamaguchi et al. 2013) and thus highlight the desire to search for potential osteoarthritis biomarkers.

The Osteoarthritis Research Society International (OARSI) together with the Foundation for the National Institutes of Health/Osteoarthritis Initiative (FNIH/OAI) biomarkers consortium are continuing to investigate osteoarthritis biomarkers. The network has developed an osteoarthritis biomarker classification, with the acronym “BIPED” that represents five categories of biomarkers: Burden of disease, Investigative, Prognostic, Efficacy of intervention, and Diagnostic (Bauer et al. 2006). There are 18 commercially available biomarkers that this network has reported, 11 of which are serum biomarkers (Kraus et al. 2017a, b) with new biomarkers identified and/or more research on the clinical utility of existing biomarkers continuously being explored. However, only a limited number of new biomarkers have been identified over recent years (Mobasheri et al. 2017). The 11 serum biomarkers that have been highlighted are COMP, hyaluronan, CPII, PIIANP, chondroitin sulfate 846 epitope (CS846), CTX-1, MMP-3, Col2-3/ 4 C-terminal cleavage product of types I and II collagen (C1,2C), C2C, nitrated epitope of the α-helical region of type II collagen (Coll2-1 and Coll2-1 NO_2_) and the cross-linked N-telopeptide of type I collagen (NTX-1). Of these to date, none fulfil all the “BIPEDs” biomarker classification criteria and some are also associated with age (hyaluronan, PIIANP and urinary C1,2C) and/or with sex (MMP-3 and hyaluronan) (Kraus et al. 2017b; Mobasheri et al. 2017). Of the biomarkers investigated, it seems that COMP, hyaluronan, and urinary CTX-11 have had the most consistent utility for the incidence and progression of osteoarthritis. Several possible osteoarthritis biomarkers are not specific or selective to joints, such as the pro- and anti-inflammatory agents, and for example, MMPs also play crucial roles in development, wound healing, and angiogenesis. Thus, the focus has currently been on the above select group of possible biomarkers for osteoarthritis only, some of which have also been used to investigate the response to exercise loading in healthy joints, predominantly in COMP, but also, for example, NTX-1, MMP-3, as well as bone biomarkers BAP and osteocalcin. However, there are possible newer technologies being developed, e.g., proteomics and the use of combinations of biomarkers for osteoarthritis (Mobasheri et al. 2017). In addition, further investigation of how mechanical loading per se regulates cartilage homeostasis (e.g., via epigenetics) may reveal novel biomarkers or therapeutic strategies to prevent cartilage degradation (Sun 2010). Thus, this review will now detail the current understanding of joint serum (unless specified) biomarker adaptation to mechanical loading, either following acute exercise or from chronic training.

## Acute serum biomarker response to joint loading

Acute loading can be considered as a single bout of exercise or physical activity that places a weight-bearing or load-bearing force on the joint. These studies have investigated resting pre-exercise baseline biomarker concentration followed by a sample taken post-exercise, and often during a period of post-exercise recovery (i.e., rest). The majority of studies have investigated patterns of cyclic loading that repeatedly loads the joint over a period of time, such as walking or running.

### The effect of shorter bouts of acute exercise on serum COMP

**Fig. 3:**
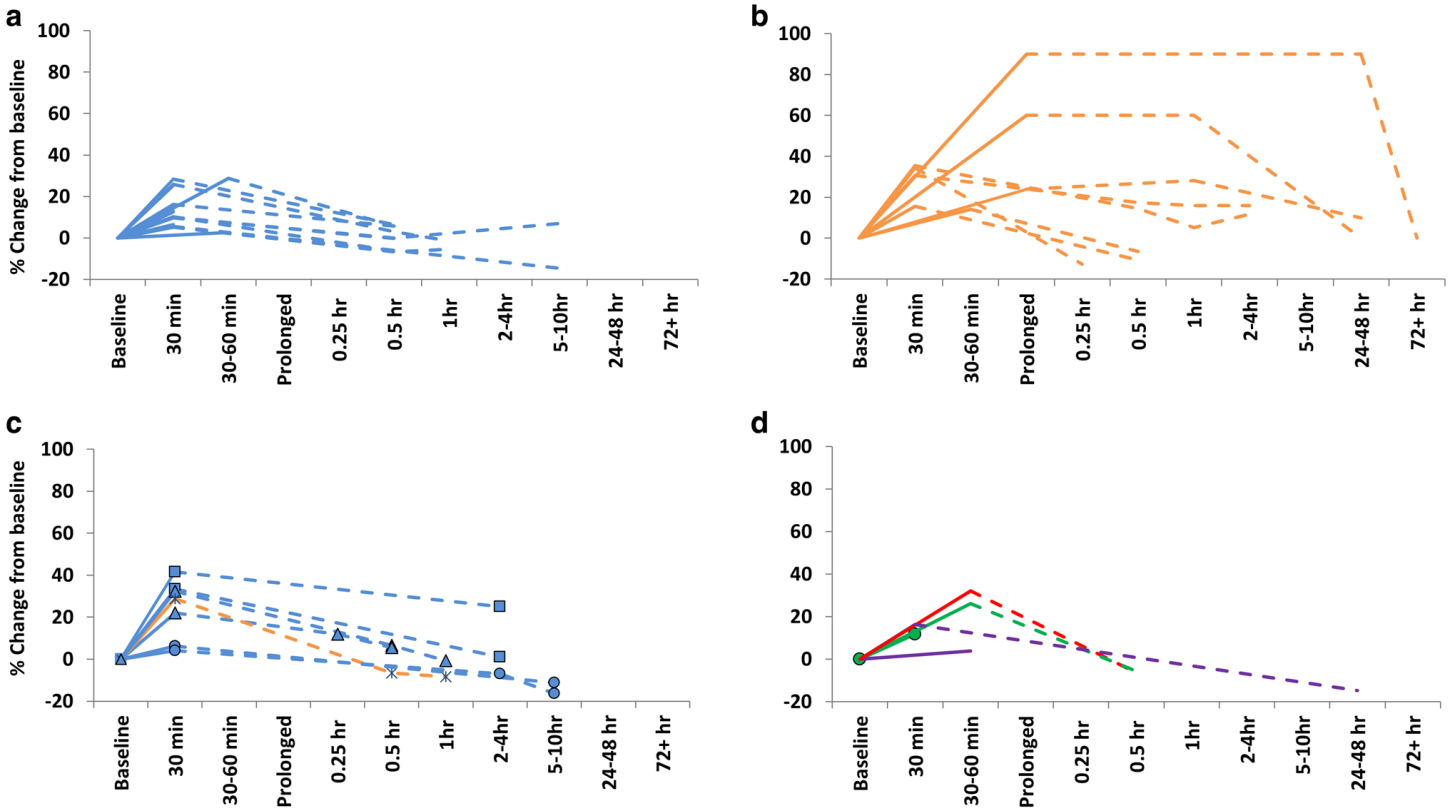
Acute exercise response of serum COMP following short (<30 min), medium length (30–60 min) and prolonged (>1 h to several hours) exercise duration (solid line) and recovery (dashed line). Data are from previous studies involving **a** walking (blue), **b** running (orange), **c** in persons that have an increase in body weight (either obese □ or simulated increase in weight △), injured Χ, or have osteoarthritis Ο, and D) following different types of exercise: cycling (red), *RT* resistance training (green), or drop jumps (purple). In some cases, the data included in these figures have been converted from its original form to enable comparison of ‘percentage change’ in serum COMP. Some data are approximate as raw data was not available

The effect of joint loading on biomarkers associated with joint cartilage has predominantly been focused on the response of the biomarker COMP. The first study to explore the acute response of COMP investigated the response before, during, immediately post and the recovery following a marathon (Neidhart et al. 2000). Results demonstrated a significant increase in serum COMP, which remained elevated for 2 h post, but had returned to baseline within 24 h. Prolonged bouts of acute running, i.e., following running a marathon has been associated with a 24–60% (Neidhart et al. 2000; Kim et al. 2009), increase in COMP. In healthy individuals, 30 min of running exercise has been associated with a 16–36% increase in COMP (Denning et al. 2014; Firner et al. 2018). Thus, COMP appears to respond in a ‘dose dependent’ fashion to acute exercise bouts (Neidhart et al. 2000; Mündermann et al. 2005; Kersting et al. 2005; Kim et al. 2009; Niehoff et al. 2010). Following shorter bouts of activity COMP usually returns toward to baseline level within 30 min of cessation of activity (Mündermann et al. 2005; Andersson et al. 2006), but may remain elevated for up to 2 days following longer bouts such as a marathon, or up to 6 days following an ultramarathon (Kim et al. 2009). Figure 3 provides a visual comparison of acute exercise studies that have investigated the effects of 30–60+ minutes of exercise on COMP.

The most common modality of exercise that has been investigated with the biomarker COMP is walking (Fig. 3a). In healthy individuals, 30 min of walking has been associated with an increase in COMP concentration of between 3–32% (Celik et al. 2013; Harkey et al. 2018). This is typically followed by a return to baseline within 30 min. Increases in joint loading, for example, by walking with a weight vest (+40% body weight) has been associated with a larger increase in COMP (+22%) compared to body weight (+10%). Similarly, walking on an incline increased COMP by + 25% compared to walking on the flat + 7% (Pruksakorn et al. 2013). In contrast to walking and running (Fig. 3a–c), the COMP response following exercise modalities such as resistance training and plyometrics is less well defined (Fig. 3d). In healthy young people, slow deep knee bends did not result in an acute increase in COMP (Niehoff et al. 2010). In contrast, drop jumps in healthy individuals have been shown to result in a significant increase (+3.8–30.9%) in COMP (Niehoff et al. 2011; Behringer et al. 2014; Harkey et al. 2018). The substantial variability in the response to drop jumps is likely to be related to protocol differences as well as the participants studied, i.e., the greatest increase (+30.9%) was found in sedentary individuals. Following a typical lower body resistance exercise bout in healthy individuals, COMP responded in a similar manner to walking exercise of the same duration (Roberts et al. 2018). Overall, the response of COMP across exercise modalities is generally similar, especially when assessing the response following walking, resistance training, and drop jumps (Fig. 3). The most prominent response is following prolonged bouts of running, suggesting that the duration of exercise may be crucial in the magnitude of change. Perhaps, future studies exploring longer bouts of different modalities of exercise can help elucidate the effect of both duration and modality on COMP response.

### The effect of both repeated and extended bouts of acute exercise on serum COMP

When considering the time course associated with acute exercise, understanding the effect of repeated bouts of exercise is also important. A study by Behringer et al. (2014) demonstrated that unlike muscle damage, COMP does not show a blunted response after two similar loading interventions. However, the COMP response to continuous joint loading over several days or weeks may demonstrate a ‘ceiling effect’. For example, in endurance, athletes completing the Transcontinental Footrace (4486 km in length) resulted in a significant COMP increase (+ 22%) at 1002 km (day 15), which subsequently remained stable for the duration of the event (Mündermann et al. 2017). Interestingly, the magnitude of increase in COMP in this study (+ 22.5%) was less than previously reported following a marathon (60%) or a single-stage ultramarathon race (90%) (Kim et al. 2009). The difference between COMP analysed following a single-stage and multistage race may represent the fact that COMP was not analysed at the same timepoint post-exercise (i.e., immediately following cessation of exercise). Therefore, it is plausible that an initial peak was present but not observed. In contrast, a 3-week multistage professional cycling race has previously been found to have a limited effect on COMP concentration (Corsetti et al. 2015). This may indicate that COMP is more sensitive to repetitive impact loading indicative of the walking / running foot strike rather than just the high cyclic joint loading of joints per se, especially as a result of longer bouts of loading. However, greater increases in COMP following cycling exercise (25 km) has been observed to that of a similar duration of running (10 km) in trained athletes (Roberts et al. 2016). The results of cartilage biomarkers following such extreme continuous loading also suggest that articular cartilage is able to adapt even to extreme joint loading. While the ability of the cartilage to adapt remains an interesting question, 12 weeks of running exercise was found to lessen the acute-loading response to walking exercise (Celik et al. 2013). Whether this represents a training adaptation requires further investigation. However, there are currently no other studies that have directly compared the acute response between trained and untrained individuals. The ability of articular cartilage to adapt to joint loading requires further investigation and cartilage biomarkers may facilitate this process.

### Possible mechanisms associated with acute changes in serum COMP

The exact mechanisms contributing to the increase in biomarker concentrations and the movement from the extra-cellular matrix into the bloodstream remains unclear. In cartilage explants, increases in mechanical loading regulate the production and turnover of cartilage macromolecules (Piscoya et al. 2005). In general, increases in COMP following loading are understood to relate to increased cartilage metabolism and/or degradation. This may relate to increases in cartilage turnover or tissue damage, i.e., the measurement of already degraded fragments, or fragments that have been degraded as a result of loading. As a key regulator of water content within articular cartilage, increases in COMP may also reflect the pressure on the articular cartilage and the movement of water from within the joint. Several studies have previously demonstrated decreases in cartilage morphology following joint loading (Eckstein et al. 2005). Moreover, changes in serum and synovial fluid COMP pre-to-post an acute bout of running have been found to be inversely correlated, i.e., the increase in COMP was correlated with the decrease in synovial fluid COMP (Hyldahl et al. 2016). This may also support the idea that acute loading facilitates the diffusion of COMP. However, the synovial fluid sample was collected at 15 min post-exercise and not immediately following cessation of the activity (as per the serum samples); therefore, we cannot discount the possibility that synovial fluid concentrations were increased immediately following activity and subsequently decreased. The relationship and kinetics between COMP at joint level and in the bloodstream remain an interesting question. While this presents a significant methodological challenge, the results of such a study would significantly benefit the understanding and future use of COMP as a biomarker. Overall, although COMP is key in the stabilisation of extra-cellular matrix, acute increases in COMP with the subsequent return to baseline post-exercise are unlikely to reflect a negative response to loading, especially in healthy individuals.

### The effect of acute exercise on additional biomarkers related to cartilage and synovium

Several prominent biomarkers related to cartilage and synovium, including structural markers, enzymes, cytokines, and inflammatory markers that can be analysed in the blood and have also been investigated in response to acute loading. These include: hyaluronan (cartilage degradation and synovitis), MMP-3 (degradation of proteoglycans and synovitis), and MMP-13 (degradation of type II collagen and synovitis) (Kraus et al. 2011). Other inflammatory biomarkers that, while not specific to the joint, may play an important mechanistic role in joint health include IL-1β, IL-6, and IL-1ra. Given the role of synovitis and inflammation in the development of conditions such as osteoarthritis and rheumatoid arthritis, these biomarkers may help our understanding of the role of loading on joint health.

Of the aforementioned biomarkers, the acute response of serum hyaluronan has received the most attention, perhaps a result of its promise as a biomarker of osteoarthritis. In healthy individuals, neither an acute bout of walking or resistance training increased concentration of hyaluronan (Roberts et al. 2018). This supports an early study by Engström-Laurent et al. (1987) who also found no evidence of a change in hyaluronan following moderate-intensity cycling, although a bout of heavy cycling exercise resulted in a significant increase in hyaluronan. Plasma hyaluronan has been shown to rise with exercise time and demonstrate an exponential increase with increasing exercise intensity in healthy individuals (Hinghofer-Szalkay et al. 2002). However, this was not a universal finding, as in healthy individuals, prolonged walking on an incline has been associated with a decrease in hyaluronan concentration compared to prolonged walking on the flat (Pruksakorn et al. 2013). The difference between the acute loading-induced response between COMP and hyaluronan indicates that these markers reflect different joint processes, with acute loading having a more pronounced effect on COMP than hyaluronan (Roberts et al. 2018). However, this may also reflect differences in the transport across the joint membrane and into the systemic circulation, and/or in the clearance of biomarkers. An additional serum biomarker that has been found to respond to acute loading is lubricin, a PRG-4 protein, encoded by the PRG-4 gene and understood to reduce the friction associated with joint movement (Jay et al. 2007). Lubricin has been shown to acutely increase following both running and cycling exercise in healthy trained individuals prior to returning to baseline within 30 min, following a similar profile as COMP (Roberts et al. 2016).

The effect of catalysers such as MMPs may also explain the response of serum joint biomarkers. MMP-3 concentration in the blood has also been shown to increase from baseline following a multi-stage ultramarathon (Mündermann et al. 2017). Interestingly, this response was correlated with the increase in COMP, indicating that MMP-3 may play a role in the degradation of non-collagenous matrix proteins such as COMP (Mündermann et al. 2017). Resistance training exercise [6 sets of 10 repetitions at ~ 75% 1-RM (one repetition maximum)] has also been found to acutely increase MMP-3 in a group of healthy untrained individuals (Urso et al. 2009); however, this response was not observed following an 8-week training programme, suggesting that training status may influence the response.

Research demonstrates that there is a significant inflammatory and cytokine response both immediately after, as well as in the period following acute exercise. Several studies have demonstrated increases in IL-6 following running [e.g., 40% increase immediately post a marathon (Nieman et al. 2001) and 160% increase following a 160 km race (Nieman et al. 2005)]. Likewise, increases in TNF-α have also been found following running (Ostrowski et al. 1998) and are understood to be intensity dependent (Kim et al. 2007). CRP has also been found to be elevated immediately post prolonged exercise, with CRP peaking the 24–48 h after running (Neidhart et al. 2000) and CRP can remaining elevated for up to 6 days following prolonged ultraendurance exercise. Overall, several pro-inflammatory biomarkers appear to peak immediately post-exercise, which depending on the activity may remain elevated for several days, prior to returning towards baseline within the hour/day post-exercise. Though, as previously mentioned, these inflammatory markers may not be joint specific.

The anti-inflammatory response appears to be characterised by a delayed peak in serum concentrations that may remain elevated for longer, returning to baseline within 24 h. Further to the systemic response, in a group of females with knee osteoarthritis, exercise was found to increase both intraarticular and peri-synovial concentrations of IL-10 (anti-inflammatory cytokine) 4 h after exercise, i.e., a local anti-inflammatory response. The anti-inflammatory response following exercise may be chondroprotective and could possible explain the beneficial effect of exercise (Helmark et al. 2010). Increases in the inflammatory marker IL-6, which is released from contracting muscles, are understood to stimulate IL-1ra and IL-10 (Petersen and Pedersen 2005). In a study exploring the response of biomarkers to marathon running, IL-1ra was correlated with COMP which may perhaps indicate that increases in cartilage metabolism or turnover may be followed by a swift counter-regulatory anti-inflammatory response. Interestingly, among individuals completing a bout of running, those with lower quality of life scores and lower activity levels experience greater increases in the inflammatory response as well as a greater increase in the collagen turnover (Cattano et al. 2017a). However, whether greater inflammation causes poor quality of life, or vice versa remains to be seen.

### The effect of acute exercise on biomarkers of bone formation and resorption

Other important joint biomarkers that have been investigated in response to acute exercise are biomarkers associated with bone metabolism, including both cell and matrix-derived markers. Key markers of bone formation include osteocalcin and BAP as well as propeptides that are cleaved during secretion by osteoblasts [procollagen type I N propeptide (PINP) and procollagen type I C propeptide (PICP)]. In contrast, TRAP-5 and telopeptides, cross-linked carboxyterminal telopeptide of type I collagen (ICTP), CTX-1 and NTX-1, cleaved during resorption by osteoclasts are key markers of bone resorption. Unlike research relevant to cartilage biomarkers, which are heavily focussed on COMP, a variety of bone biomarkers have been utilised to investigate the response to acute loading. A number of these bone biomarkers have clinical usefulness and are reported to be associated with bone microarchitecture, predict fracture risk, and have been associated with osteoarthritis. However, unlike research on cartilage biomarkers, there is currently limited research studies exploring the effect of walking on bone biomarkers. In healthy men, 30 min of walking did not change serum bone BAP or osteocalcin concentration (Welsh et al. 1997). Similarly, Rudberg et al. (2000) demonstrated limited effect of acute jogging (4–7 km run) in healthy young women on all osteocalcin and BAP isoforms, except for BAP B2 isoform which demonstrated a small increase. An acute bout of resistance training did not change BAP or osteocalcin biomarker concentrations in healthy males, or PICP in sedentary healthy males (Whipple et al. 2004). In contrast, an incremental cycling test (20–32 min in duration) significantly increased all bone BAP isoforms (B, B1, and B2) among postmenopausal women, returning towards baseline after 20 min (Rudberg et al. 2000). However, changes in concentration of BAP have been found to be significantly decreased 2- and 3-day post-acute exercise (Ashizawa et al. 1998), while PICP decreased similarly 24 h post a short bout of running in trained female runners (Brahm et al. 1996). Gombos et al. (2016) investigated the direct effects of physical training on markers of bone metabolism and sclerostin concentrations in 150 otherwise healthy female participants diagnosed with osteoporosis or osteopenia. The study concluded that resistance exercise reduced the concentrations of CTX-1, a marker of bone resorption, but walking did not. Overall, the effect of exercise on bone biomarkers may be dependent on the individual and the modality of exercise. Compared to short exercise bouts, a significant decrease in osteocalcin was demonstrated immediately post a marathon (Malm et al. 1993). Likewise, BAP also decreased immediately postmarathon in males, but not female runners. Indicating sex-based differences may exist between bone formation biomarkers following marathon running. Moreover, in male runners, prolonged exercise (a marathon) was associated with an immediate decrease in PICP, returning towards baseline 1-day post, prior to significantly increasing beyond pre-exercise levels 3-day post (Langberg et al. 2000). In male runners that completed a 245 km ultramarathon, osteocalcin and BAP have been found to decrease immediately after, and remain decreased 24 h post, suggesting a suppression in bone formation (Mouzopoulos et al. 2007). The difference between the bone formation biomarkers in these two studies may reflect the difference in running duration.

Markers of bone resorption demonstrate considerable variability when assessed post-acute exercise. For example, the immediate effect of acute exercise on TRAP-5 demonstrated no change following high-intensity resistance training (Bemben et al. 2015) and following plyometric training (Rogers et al. 2011). Similar results have been reported in ICTP concentrations following Wingate tests (Kristoffersson et al. 1995) and 30–40 min of jogging (Rudberg et al. 2000). However, decreases in TRAP-5 have been observed 15–30 min postresistance training and plyometric training (Rogers et al. 2011), while CTX-1 and NTX-1 have also been found to acutely increase 30 min post 1 h of cycling (Guillemant et al. 2004) and 1 h post 45 min of resistance exercise (Whipple et al. 2004). There is some evidence that increases in ICTP occur immediately post following prolonged loading such as marathon running (Langberg et al. 2000); however, as demonstrated by the non-significant increase in ICTP following ultrarunning (Mouzopoulos et al. 2007), this is not conclusive. Overall, increases in bone resorption markers are more consistently observed in the day postloading, e.g., 24 h post (Thorsen et al. 1997) and 2-day post (Brahm et al. 1996). Underlying mechanisms for the increases in osteoclast activity is poorly understood.

### Summary of the biomarker response to acute exercise

This review demonstrates that the response of several biomarkers has been investigated following bouts of acute exercise, including structural markers, enzymes, cytokines, and inflammatory markers, though most have focused on COMP. These biomarkers and their response may provide an indicator of the effect of acute joint loading on cartilage, bone and synovium, as well as the joint as a whole. The response of biomarkers to acute loading would indicate that joint structures are dynamic, with even a modest bout of acute walking resulting in increases in markers associated with cartilage metabolism (Fig. 3). Unsurprisingly, prolonged loading through exercise such as marathon or ultramarathon running elicits a heightened response in cartilage metabolism/degradation (Fig. 3b) and inflammation when assessed immediately post-exercise. However, cartilage biomarkers return to baseline shortly following recovery, likely indicating that these are transient responses and not indicators of degradation. In contrast, biomarkers of bone metabolism appear less sensitive to immediate change, and instead, changes are more evident in the hour or day following exercise, i.e., during recovery. This is unsurprising, given that changes in bone cell function are likely to occur up to 24 h after exercise (Banfi et al. 2010). There is also widespread variation in bone biomarkers and the response and recovery to loading, which may reflect the ‘hectic’ activity of bone, but as well as other factors, including differences between studies, exercise, as well as inter-individual differences. Overall, research on the acute response of joint biomarkers remains limited, with the vast majority of studies investigating healthy individuals, and only a limited number investigating different modalities of exercise.

## Serum biomarker response to exercise training

### The effect of exercise training on biomarkers of cartilage metabolism

Exercise training is the process of performing repetitive physical activities or body movements to improve endurance, flexibility, or muscular strength. Biomarkers of joint metabolism have been used to determine the effects of chronic exercise training on the joint. However, there are limited studies of the effect of exercise training on COMP and other biomarkers indicating cartilage metabolism. Studies of COMP in healthy individuals have investigated the effects of vibration training (Liphardt et al. 2009), collegiate soccer training (Hoch et al. 2012), and swimming and cycling training (Celik et al. 2013). In the study by Liphardt et al. (2009), the effects of immobilisation did not appear to be offset by vibration training twice per day for 2 weeks. COMP reduced significantly in both ‘bed rest’ and ‘bed rest with vibration training’ groups, leading to the suggestion that increases in COMP requires movement as well as loading per se. Hoch et al. (2012) examined COMP in collegiate soccer athletes over a 4-month athletic season, with their data, indicating that resting COMP levels increased over the season. However, the differences in COMP levels did not reach minimally detectable change values [the minimal change that falls outside the measurement error in the score of an instrument used to measure a symptom (Kovacs et al. 2008)]. Despite this, results indicated that fluctuations in COMP occur during a competitive season and must be taken into consideration for future biomarker studies. In a randomised controlled trial, the response of COMP was assessed after 30 min of walking prior to, and following 12 weeks of cycling, swimming and running training (Celik et al. 2013). Post-training measurements revealed no change in resting measurements following 12 weeks of exercise training. However, as previously discussed, training did influence the COMP response to walking exercise in the running group, i.e., 12 weeks of regular, weight-bearing, high-impact physical exercise (running) decreases the deformational effect of walking activity, offering evidence of functional adaptation of articular cartilage to specific environmental requirements.

Although not specifically assessing the effects of an exercise training programme, the following studies were conducted in trained athletes competing in events involving sustained exercise over multiple days. Over the course of a 3-week stage race involving elite cyclists, among the cartilage degradation markers, only CTX-11 was decreased, while COMP remained unchanged. Bone marker concentrations in both serum and urine were slightly but significantly decreased. The changes in bone and cartilage turnover indexes were correlated with the indexes of physical effort and energy consumption. Overall, it appeared that strenuous physical effort, in the absence of forces generated through impact with the ground (i.e., ground-reaction forces), slowed bone metabolism and did not affect cartilage turnover (Corsetti et al. 2015). In athletes competing in a multi-stage ultramarathon consisting of 4486 km over 64 running days without any rest days, Mundermann and colleagues (2018) analysed cartilage biomarker levels in the serum within 4 days before the race and on days 15, 31, 47, and 58 during the race. COMP, MMP-9, and MMP-3 changed significantly over the race, with concentrations increasing during the first measurement interval (after 15 days, at 1002 km) by an average of 22.5% for COMP, 22.3% for MMP-3, and 95.6% for MMP-9 and then remained stable throughout the remainder of the event. MMP-1, C2C, CPII, and C2C:CPII did not change over the race.

### The effect of exercise training on biomarkers of bone formation and resorption

Studies have also investigated the effects of exercise training on serum markers of bone metabolism. One previous study measured bone formation and bone resorption markers in trained participants before and following 5 weeks of training on 5 days per week, for 2 h (Eliakim et al. 1997). They found that bone formation markers BAP, osteocalcin, and PICP increased significantly in the training group participants, with bone resorption markers pyridinoline and CTX-1 not modified and NTX-1 decreasing. In another study, participants were grouped into either an 8-week training intervention involving endurance running, or an 8-week anaerobic training intervention involving sprinting and weightlifting, or a control group (Woitge et al. 1998). Bone formation and bone resorption biomarkers were measured at week 4 and week 8. Similarly, BAP and osteocalcin, despite a significant decrease at week 4, increased overall over the training period. Pyridinoline and deoxypyridinoline also increased at week 8.

Several studies have examined the effects of long-term training (more than 6 months) and competition on bone markers and these have been summarised by Banfi et al. (2010). In rowers, an increase in osteocalcin was observed (Jürimäe et al. 2006). Decreases in BAP were observed in triathletes (Maïmoun et al. 2004), whereas an increase in BAP was observed in sedentary females who completed daily walking or daily walking and jumping, and were assessed 1 year later (Shibata et al. 2004). In addition, a study explored the seasonal variation of bone markers in elite female skiers and concluded that the formation markers, BAP, and osteocalcin and the resorption marker TRAP-5 significantly increased from the end of the pre-competitive season to the end of the competitive season, while CTX-1 showed no significant changes (Lombardi et al. 2011). The authors suggested that this was probably linked to the highly demanding period of competitions when bone is more stimulated by mechanical forces through weight-bearing exercise, again, indicating that competitive seasonal variation needs to be considered when examining the effect of exercise on bone biomarkers.

### The effect of exercise training on inflammatory biomarkers related to the joint

Regular exercise training is often considered to have anti-inflammatory properties and may consequently have a role in joint health. Several observational studies have documented the negative correlation between self-reported physical activity level and markers of inflammation including CRP, IL-6, and TNF-α, which have also been documented independent of obesity (Colbert et al. 2004; Pitsavos et al. 2005). A recent systematic review of randomised control trials also indicated that aerobic exercise training may have a positive effect on reduction of CRP, TNF-α, and IL-6 in middle-aged and older adults (Zheng et al. 2019). In contrast to aerobic training, fewer studies have investigated resistance training exercise. In an early study, 12 weeks of progressive high-intensity resistance strength training was found not to affect IL-1β, TNF-α, or IL-6 production (Rall et al. 1996). Similarly, 12 weeks of resistance training did not significantly reduce CRP, IL-6, or IL-8 (Kohut et al. 2006). This suggests that aerobic training may be key to obtaining the beneficial effects of exercise training. In older individuals (i.e., individuals that may be at risk of joint conditions), a recent 12-month randomised controlled trial comparing a physical activity (consisting of a combination of aerobic, strength, balance, and flexibility exercises) to a non-exercise control group showed a significant decrease in only two biomarkers (IL-6 and IL-8) in the physical activity group, compared to the non-exercise intervention (Beavers et al. 2010). Interestingly, there was a greater effect among individuals in the physical activity group with a higher baseline inflammatory status (IL-6) (Nicklas et al. 2008; Beavers et al. 2010).

### Summary of the biomarker response to exercise training

While cartilage biomarkers do vary across the course of a competitive season, changes in baseline cartilage metabolism following shorter training studies are less clear. Perhaps, training influences the acute deformation of cartilage in response to exercise, rather than resting cartilage metabolism per se. While exercise training consistently increases markers of bone formation, bone resorption may also increase following periods of strenuous training. Finally, the inflammatory response to training, which may also have a key role in joint health, indicates that regularly engaging in moderate levels of aerobic exercise training may be most beneficial, with the greatest effect among individuals with higher baseline inflammation.

## The serum biomarker response to acute and chronic exercise training among individuals with pathological conditions

**Fig. 4:**
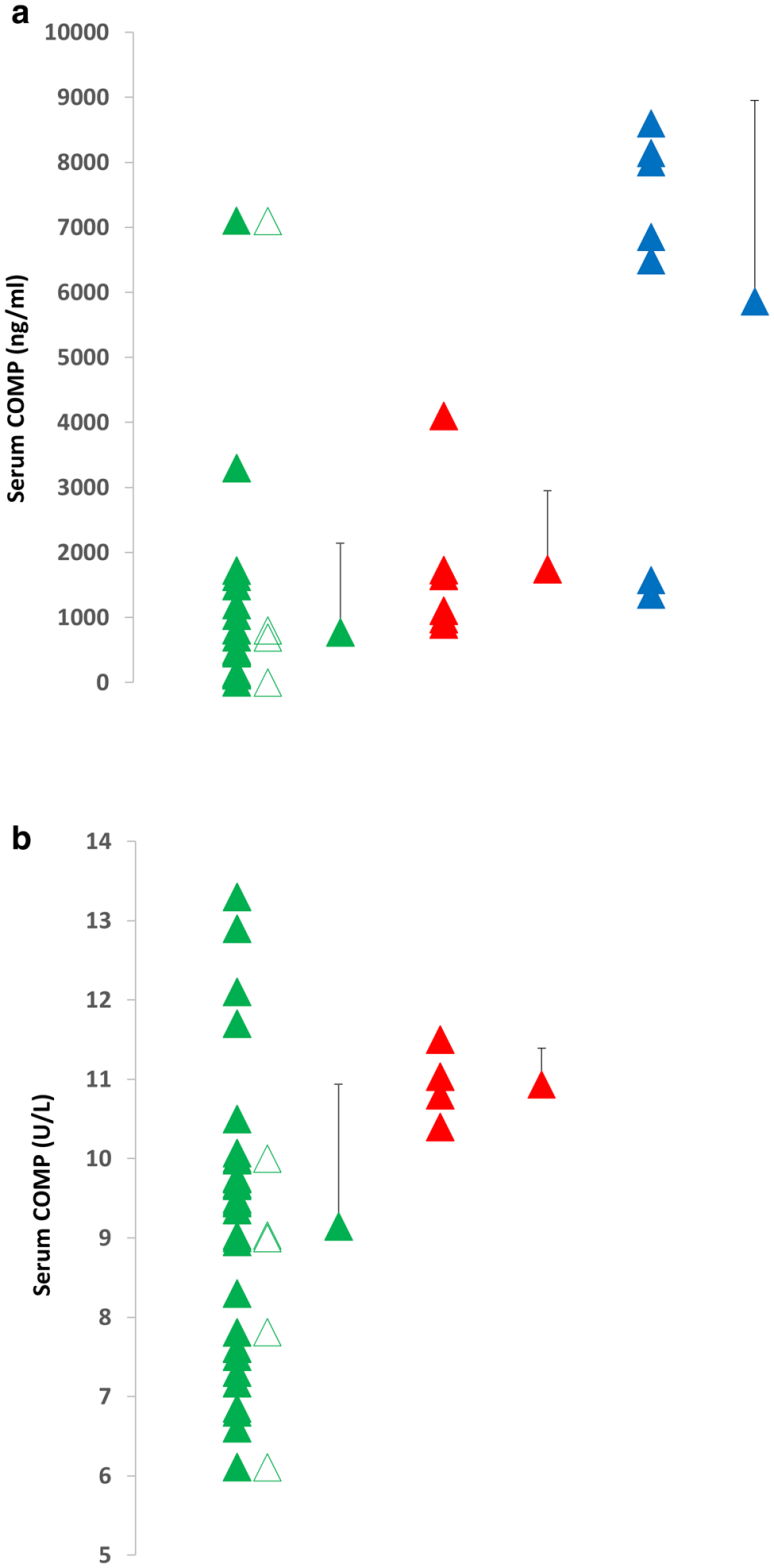
Differences in baseline serum COMP between groups as obtained from mean/medians reported in the previous studies; healthy (green), trained (open green), osteoarthritis (red), and injured (blue). Data from previous studies expressed as **a** ng/ml or **b** U/L, with mean and SD of data presented

As mentioned earlier, a key driver behind ascertaining joint serum biomarkers is to determine the degradation status of joints if this can be predicted prior and/or assist in treatment. Common joint biomarkers for osteoarthritis are listed in Fig. 2 and have been reviewed elsewhere (Henrotin et al. 2016; Cattano et al. 2017b; Kraus et al. 2017b; Bay-Jensen et al. 2018). In summary, many cartilage, synovium, bone, and inflammatory markers have been shown to be altered in people with osteoarthritis at rest leading to their possible utility in a clinical context, although no clear osteoarthritis biomarkers have yet been determined (Kraus et al. 2017b; Bay-Jensen et al. 2018). A recent systematic review assessing COMP, CTX-11, and MMP-3 in knee and hip osteoarthritis patients found that COMP and urinary CTX-11 can distinguish osteoarthritis patients from healthy, with COMP effectively able to predict osteoarthritis progression (Hao et al. 2019). Resting or baseline biomarker concentrations can provide an indicator of the overall physiological milieu of the joint. Although traditionally biomarkers have been utilised to investigate osteoarthritis, they may also have a role in determining the status of healthy joints and to help identify adaptation postinjury, or early age-related changes. For example, concentrations of COMP are generally elevated among individuals with osteoarthritis or joint injury compared to healthy individuals (Fig. 4). In relation to the acute and chronic exercise effects on these biomarkers in people with osteoarthritis, there is less clarity. The acute COMP response has previously been correlated with a decrease in cartilage volume (Kersting et al. 2005) and changes in cartilage thickness over a 5-year period (Erhart-Hledik et al. 2012). Therefore, it has been postulated that the COMP response to loading may provide a measure of the health of joint cartilage and help identify abnormalities or disease such as osteoarthritis. Studies that have investigated the response of COMP to acute loading can be found in Fig. 3c, d.

### The effect of acute exercise on the biomarker response among individuals with pathological conditions

Overall, acute joint loading in joints with recognised osteoarthritis has previously been investigated, albeit not extensively. For example, following 30 min of walking COMP concentrations have been found to acutely increase between 4–6.3% in individuals with osteoarthritis (Mündermann et al. 2009; Erhart-Hledik et al. 2012). One study that directly compared the response of 30 min of walking (self-selected speed) in a group of overweight individuals with and without osteoarthritis found a similar acute increase (+ 6.3% and + 5.6%, respectively). Concentrations returned towards baseline in both groups within 30 min (Mündermann et al. 2009). However, in healthy individuals, the response of COMP concentrations to 30 min of walking has varied in magnitude (+5.6–31.9%; Mündermann et al. 2009; Celik et al. 2013). Differences in the response may relate to differences in baseline values among individuals with osteoarthritis, which are typically elevated, as well as factors such as differences in the intensity of walking. Similarly, Andersson et al. (2006) demonstrated that 60 min of lower body circuit training session in patients with knee osteoarthritis significantly increased COMP concentrations which subsequently normalised after 30 min. However, increases in response to resistance exercise are not a universal finding among individuals with joint conditions. In patients with rheumatoid arthritis, acute lower body resistance exercise involving three sets of eight repetitions did not result in a significant increase in COMP (Law et al. 2015). Studies investigating the acute COMP response among individuals with joint injury are also limited. An experimental study exploring the effect of anterior knee pain on the COMP response to 30 min of running did not alter the response compared to the control trial (Denning et al. 2014). While similarly, a recent study previously demonstrated that an acute bout of moderate-intensity running resulted in comparable biochemical responses between young, active individuals in a high-risk postinjury population and matched healthy controls (Cattano et al. 2017a). In patients with rheumatoid arthritis, however, moderate-intensity cycling elicited a large increase in hyaluronan (Engström-Laurent et al. 1987). The exercise-induced increase in rheumatoid arthritis patients was related to synovitis mass, suggesting that the concentration of hyaluronan in the joint may be related to joint inflammation. However, individuals with lower activity and quality-of-life scores experienced greater collagen turnover and inflammation following running (Cattano et al. 2017a). Moreover, as documented in Fig. 4, baseline COMP may be elevated among injured individuals or with osteoarthritis compared to healthy individuals. It is currently unknown whether exercise in individuals with elevated levels of baseline COMP (i.e., following acute injury or with joint disease) is associated with positive or negative changes to joint tissues. Therefore, attempting to characterise the ‘normal’ acute response to loading may offer the opportunity to identify an ‘abnormal’ loading response and provide guidance for optimal exercise prescription and safe return to activity.

### The effect of exercise training on the biomarker response among individuals with pathological conditions

Studies investigating the joint biomarker response to chronic exercise training are also limited in populations with injury or joint degradation. Three studies have assessed the effects of long-term exercise training in people with osteoarthritis (Andersson et al. 2006; Chua et al. 2008; Petersen et al. 2010) and two studies that have investigated the effects of exercise training in people with rheumatoid arthritis (the RAPIT study; de Jong et al. 2008; Law et al. 2015). Andersson et al. (2006) monitored levels of COMP during a randomised controlled trial of physical exercise training (twice per week for 24 weeks) versus standardised rest in individuals with symptomatic and radiographic knee osteoarthritis. Median COMP values in samples obtained prior to exercise or rest at baseline and after 24 weeks did not change between the start and end of the study. The effects of an exercise and weight loss intervention in overweight and obese adults with osteoarthritis of the knee and found that the serum levels of hyaluronan, COMP, and TGF-β remained relatively stable during the 18-month intervention period, while there was an overall slight decline in antigenic keratan sulfate (Chua et al. 2008). The levels of serum COMP and urine CTX-11 in patients with osteoarthritis of the knee in response to muscle strength training in combination with treatment with glucosamine, ibuprofen or placebo has also been investigated (Petersen et al. 2010). Training per se did not seem to induce changes in circulating levels of markers for cartilage degradation, whereas analysis of the specific medication effect revealed that glucosamine decreased COMP significantly compared to treatment with placebo or ibuprofen. However, it is not possible to elucidate whether this reduction in COMP levels transforms into a better clinical status in these patients from this data. In the study by Law et al. (2015), nine stable rheumatoid arthritis patients completed an 8-week combined and progressive exercise programme designed to improve aerobic fitness and lower body strength. Participants were assessed at baseline and 1 h post-exercise at weeks 0, 4, and 8. No changes in post-exercise COMP, synovial inflammation, or CRP were observed over the 8-week intervention. This research suggests that, in patients with inactive rheumatoid arthritis, continued intensive exercise training was not associated with changes in cartilage metabolism. Moreover, it appears that the acute response to exercise is not affected by continued exercise training, similar to findings in healthy individuals (Celik et al. 2013). Finally, de Jong et al (2008) studied people with rheumatoid arthritis and found that after 3 months, the mean COMP level increased slightly with in the high-intensity weight-bearing exercise group and decreased in the usual care group. However, these changes from baseline and the mean difference in change between the groups were not statistically significant.

### Summary of the biomarker response to exercise among individuals with pathological conditions

Overall, studies are again largely limited to COMP, and presently, we are unable to determine whether the acute exercise response for this, or other biomarkers, are outside of the normal range for healthy joints. At rest, and despite large variation, Fig. 4 demonstrates that individuals with osteoarthritis or injured/previously injured generally have higher resting values of COMP compared to healthy individuals. However, from the available data, results indicate that the acute response in osteoarthritis is largely unchanged compared to healthy individuals, indicating that the exercise interventions investigated appear to be safe and do not result in any acute adverse alterations in cartilage metabolism. This is somewhat reassuring, given that exercise is typically utilised as a therapeutic tool following both injury and among individuals with osteoarthritis and rheumatoid arthritis. To date, training studies have not provided any indicators of an adverse effect of training among individuals with either osteoarthritis or rheumatoid arthritis. Further work, possibly utilising a variety of biomarkers, is required to identify whether biomarkers and the response to both acute and chronic exercise can be utilised to tailor exercise interventions and monitor joint health.

## Future recommendations

Several studies have determined that serum biomarkers respond to acute loading, with the response and time course possibly dose dependent. Factors that may influence the response include age, bodyweight, and joint injury or degeneration. Future studies must continue to characterise the normal biomarker response in both the healthy and compromised joint to determine whether an ‘abnormal’ response or ‘threshold’ exists. Given that moderate loading is considered chondroprotective therapy (Sun 2010), biomarkers may assist in determining the most beneficial stimulus for joint health, i.e., not underloading or overloading.

In relation to chronic exercise training, it is important to note that studies have been limited to people with a similar baseline COMP. Therefore, the effect of continued, high-intensity exercise on COMP levels of patients with active disease and patients with ‘high’ COMP levels is also an area for additional investigation. It is also necessary to reliably determine the effects of longer term exercise programmes (i.e., more than 3 months) on these outcome variables. This could perhaps provide health professionals with further supporting information enabling them to recommend exercise with further credence and address patient concerns relating to joint health. Further research is also required to address the limitations associated with the range of patients to whom these results can be applied.

Currently, studies of both acute and chronic exercise have been limited to COMP (see Fig. 2); however, other promising biomarkers exist, including a number that have been identified as biomarkers of osteoarthritis. Several of these have been shown to respond to acute exercise. Utilising additional biomarkers, or a cluster of biomarkers, as proposed for osteoarthritis (Mobasheri et al. 2017), may provide a better indicator of the joint following acute and chronic loading.

Finally, a key factor for the use of biomarkers is the process of standardisation. There are difficulties with direct comparison between studies due to large variability in reported baseline values, even in a relatively homogeneous group of healthy individuals. This is further compounded by a difference in the units of measurement between studies, i.e., ng/ml and U/L. Variability may also be explained by the use of a variety of different ELISA kits to measure biomarkers. As previously highlighted in assays intended to assess collagen biomarkers, these may potentially be measuring slightly different isotopes (Birmingham et al. 2007). There is also the issue of lack of standardisation in the timing of blood draws between studies; including the specified period of unloading and rest prior to the draw, as well as the time of day of each draw. The differences between protocols is also at the post-exercise draw, which has been reported between immediately and 30-min post. Given that following 30 min is often sufficient to see values return to baseline levels, any potential effect of acute exercise may be lost in these studies (e.g., Kersting et al. 2005; Niehoff et al. 2010). Consequently, this makes meta-analyses very challenging. Furthermore, the impact of standardisation for plasma volume may be particularly important during longer/high-intensity bouts of exercise, i.e., when plasma volume is more likely to change. Although several studies have applied corrections when exploring biomarkers associated with bone (Rogers et al. 2011; Bemben et al. 2015), this is not applied throughout the studies. Awareness of additional factors such as the influence of age, sex, and race on biomarker concentrations is also warranted when exploring serum or urine biomarkers (Abramson and Krasnokutsky 2006).

## Conclusions

The majority of research investigating acute loading has focused on the serum COMP response in healthy young individuals. Results suggest that acute loading transiently increases cartilage metabolism without any lasting deleterious changes, evidence of cartilage degradation, or association with osteoarthritis. However, more research is needed to explore the biomarker response to exercise following joint injury. Moreover, while the type of exercise appears to be less important for the nature or magnitude of the observed response, prolonged exercise may result in higher responses in serum COMP that can remain elevated for several days. Other biomarkers, such as those of bone metabolism, synovium, and inflammation, are less well defined. The use of multiple biomarkers that provide information on the various components related to joint health, as observed from osteoarthritis research, and which encompass markers of synthesis, degradation and inflammation, still warrant further investigation. Whilst our understanding of the response to acute and chronic loading is developing, further research is also required to establish the normal biomarker concentration range for healthy joints, including concentrations at rest, and in response to acute exercise, as well as following chronic training. Perhaps such information could inform the safety of both acute and chronic loading for joint structure as well as overall joint health. Finally, for this field to move forward, an improved understanding of the relationship between serum concentrations and changes at the joint level is required, including structural changes. This should be combined with improvements in assessment standardisation and biochemical analysis techniques. For studies exploring the effect of acute loading on serum (joint tissues) COMP concentrations, we recommend a standardisation of 30 min seated rest prior to baseline/resting blood draw, with subsequent blood draws obtained immediately post-exercise and at 30 min post-exercise.

## Abbreviations

BAP: Bone alkaline phosphatase
C2C: Collagen type II cleavage product
COMP: Cartilage oligomeric matrix protein
CPII: C propeptide of type II
CRP: C-reactive protein
CS846: Chondroitin sulfate 846 epitope
CTX-1: C-telopeptide of type I collagen
CTX-11: C-telopeptide of type II collagen
ICTP: Cross-linked carboxyterminal telopeptide of type I collagen
IGF-1: Insulin-like growth factor
IL: Interleukin
MMPs: Matrix metalloproteinases
NTX-1: N-telopeptide of type I collagen
PICP: Procollagen type I C propeptide
PIIANP: N-terminal propeptide of collagen IIA
PINP: Procollagen type I N propeptide
PRG-4: Proteoglycan-4 (lubricin)
TGF-β: Transforming growth factor beta
TNF-α: Tumor necrosis factor
TRAP-5: Tartrate-resistant acid phosphatase 5b
VEGF: Vascular endothelial growth factor
